# The Effect of Caries on the Chewing Ability of Children: A Scoping Review

**DOI:** 10.1055/s-0042-1758066

**Published:** 2022-12-13

**Authors:** Taufan Bramantoro, Wahyuning Ratih Irmalia, Cornelia Melinda Adi Santoso, Nor Azlida Mohd Nor, Haryono Utomo, Aulia Ramadhani, Risma Aprinda Kristanti, Alexander Patera Nugraha

**Affiliations:** 1 Department of Dental Public Health, Faculty of Dental Medicine, Universitas Airlangga, Surabaya, Indonesia; 2 Indonesian Health Innovation and Collaboration Institute, Surabaya, Indonesia; 3 Faculty of Public Health, University of Debrecen, Debrecen, Hungary; 4 Department of Community Oral Health and Clinical Prevention, Faculty of Dentistry, University of Malaya, Kuala Lumpur, Malaysia; 5 Department of Forensic Odontology, Faculty of Dental Medicine, Universitas Airlangga, Surabaya, Indonesia; 6 Student of Doctoral Program, Faculty of Dental Medicine, Universitas Airlangga, Surabaya, Indonesia; 7 Department of Biomedical Sciences, Medical Study Program, Faculty of Medicine and Health Sciences, UIN Maulana Malik Ibrahim Malang, Malang, East Java, Indonesia; 8 Department of Orthodontics, Faculty of Dental Medicine, Universitas Airlangga, Surabaya, Indonesia

**Keywords:** caries, children oral health, mastication, masticatory performance, medicine

## Abstract

Childhood caries might have several effects on the children's general health and growth, including chewing ability. This study aims to identify the evidences found regarding the effect of caries on the chewing ability of children through a scoping review. A scoping review literature search was performed in three databases (Scopus, PUBMED, and Web of Science) without restricting the publicized year. The selected articles were using human as its subjects and aiming to analyze the effects of caries on mastication ability in children. Ten articles matched the inclusion criteria of this review. All the articles suggested a deleterious effect of caries on masticatory performance, maximum bite force, swallowing threshold, and even masticatory behavior. Two of them stated that the effect was reversible by giving dental treatment. This scoping review concludes a negative effect of caries on the children's chewing ability.

## Introduction

Dental caries is a chronic dental health problem which is, in fact, preventable. However, global reports state no improvement in children oral health over the past decades. 1 According to a study, untreated dental cavity might be a caries predictor in the permanent dentition. Meanwhile, most parents/caregivers belief that caries denotes an acute disease that should be noticed only when there is a visible cavity or when the pain arise. 2 Unfortunately, when the dental pain arises, it might had already influenced the children's development both psychologically and physically, and their school and daily life achievements as well. 3 Aside from pain, caries might also affect many other physiological functions of the body, including nutrition intake, chewing difficulty, delayed growth, 4 5 cognitive function, 6 and oral health-related quality of life (OHRQoL). 7 Prior to understanding those impacts, a better understanding on the effect of caries to mastication ability and performance is required.

Mastication poses the important stimulus for craniofacial growth and development, also in digestive process to obtain the required nutrition for health maintenance and growth. 8 The thickness of occlusal and near contact area relates to the efficiency of mastication. 9 10 An untreated caries means reduction in occlusal and proximal area, which are important for shredding foods. Moreover, pain might also arise as bolus makes contact with the dentinal tubules or the exposed pulp chamber. Thus, individual with worse caries might have worse mastication ability. 8

The effect of caries to chewing ability on children has become interesting over decades. Early childhood denotes a crucial time to acquire mastication skill. Food diversity and textures is considered as crucial factors for mastication skill maturation. 11 Thus, the effect of caries to chewing ability is naturally contrast to the adult. There are a couple of studies that focus on this area. Meanwhile, to the best of our knowledge, a study providing the whole picture of the effect of caries toward masticatory performance on children has not yet been available. Thus, this study aimed to identify the evidences found regarding the effect of caries on the chewing ability of children..

## Methods

This review has been registered in PROSPERO with ID CRD42022309417. In the interest of answering the research question—“What is the effect of caries to the mastication ability of children?”—a review was performed on some particular selected articles. Those articles' eligibility was decided based on the following criteria:

1. All types of studies (observational and experimental studies); review articles are not included.
2. Written in English.
3. Studies with children and toddler as the subjects.
4. Studies that performed all types of masticatory evaluation on children and toddler with or without caries.
5. Outcome of study: mastication ability.

A sequential group discussion was performed in order to match the perception of operational definitions of variables and data extraction. Shall any problems and questions arise throughout the data extraction it will be discussed further by all the authors.

### Search Strategy

Articles for the current review were acquired from databases (PUBMED, SCOPUS, and Web of Science), considering that they hold the most databases for biomedical and pharmaceutical literatures. Aside from that, PUBMED is known as the main tool to explore any literature relevant to biomed. The literature search was performed without restricting the year of publication, until January 13, 2022 with phrases complementary to caries, primary teeth caries, children, toddler, mastication performance, and mastication ability.

### Study Selection

Data selection was performed by two authors independently, followed by identifying and removing duplicates. Articles screening was subsequently performed according to the inclusion criteria. Hereafter, full texts were further assessed for its eligibility. The assessment result from the two authors was combined. The critical appraisal for included studies was performed using the Joanna Briggs Institute Critical Appraisal tools for cohort, cross-sectional, and quasi-experimental studies. A discussion forum was opened as soon as any disambiguates or disagreements arise. The data of the included articles were recorded and tabulated.

## Result

**Fig. 1:**
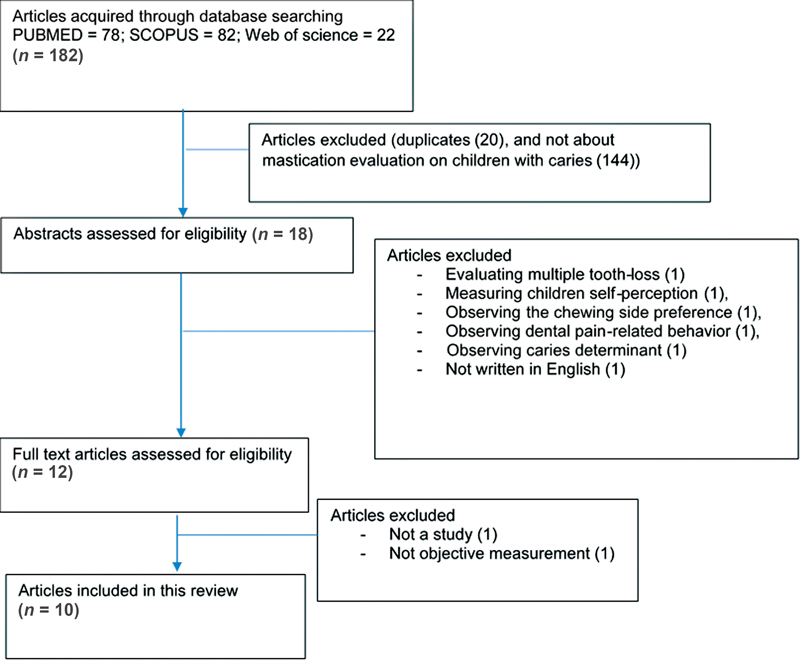
Flowchart diagram of the research search.

Following database search, a total of 182 articles were acquired and screened for its eligibility based on its title. As much as 20 duplicates were removed, and 146 articles were also removed because of different topic. Then, 18 articles were further assessed based on its abstract, eliminating 5 articles for having unmatched aim, and 1 article for not written in English. A number of 12 articles in full text were further assessed for its eligibility, removing 2 articles. In the end, a total of 10 articles were included in this study. The Preferred Reporting Items for Systematic Reviews and Meta-Analyses flow diagram of the research search is shown in Fig. 1 for this analysis.

### Characteristics of the Studies

The effect of caries toward chewing ability in children was first published in 2004, and the most publication was recorded from 2016 to 2020, with eight articles being published over that period. The included studies were from Asia, Europe, and South America. The largest portion of publication in this study were from South America (40%), followed by Asia (30%) and Europe (30%). Most of the articles were came from Brazil (4), and two articles came from France, one from Saudi Arabia, one from India, one from Singapore, and one from Turkey. The average respondents used for those studies were 171, with the maximum number being 676 and the minimum number being 25 respondents. Seven of the 10 studies were cross-sectional observational studies, two were cohort studies, and one quasi-experimental study. The subject of the studies was varied between primary and mixed dentitions, with sample age ranging from 3 to 12 years old. Only one study used target in the phase of early permanent dentition. The included articles varied from cross-sectional, cohort, and quasi-experimental studies.

### Methodological Quality

1. The cross-sectional studies

The cross-sectional studies are summarized in Table 1 . Most of the studies used the Decay, Missing, and Filling (DMFT) index 7 11 and International Caries Detection and Assessment System 11 12 13 to assess caries condition. Other clinical oral condition assessment was performed using Caries Assessment Spectrum and Treatment, 16 radiographic imaging, 14 and manually counting the number of carious lesion and plaque index. 15 Some studies also evaluated occlusion using the International Orthodontic Treatment Needs (IOTN) index 11 13 and Dental Aesthetic Index, 7 number of masticatory units, sucking habits, 13 orofacial dysfunction using the Nordic Orofacial Test-Screening (NOT-S) questionnaire, the presence of abscess, or other oral condition resulting from untreated caries using the PUFA index. 11 Some studies also collected other data that are expected to affect masticatory function, such as anthropometric measurement with body mass index (BMI) 12 or body weight and height. 14

**Table 1:** The summary of the cross-sectional studies

No.	Authors and journal detail	Subject	Sample size	Study exposure	Intervention	Outcome
1	Consolação Soares et al; Clinical Oral Investigation (2017) 21:159–166 12	Children 3–5 years old	257	Anthropometric measurement with BMI calculation (WHO)clinical oral examination using ICDASbreathing evaluation	Food consistency evaluation by dietary log filled by parents/caregiver, and masticatory performance evaluation	Higher BMI, greater number of caries, and higher frequency of sweet beverage consumption negatively affect the preschoolers masticatory performance
2	Tsai; The Journal of Clinical Pediatric Dentistry (2004); 28(2):139–142 15	Children 3–5 years old	676	Clinical oral examination by counting number of carious lesion and plaque index	Measuring occlusal bite force using occlusal force meter	The number of carious lesion and plaque index score negatively correlates the maximum bite force
3	Kaya et al; European Journal of Paediatric Dentistry (2017); 18(1): 116–120 14	Children 12–14 years old	50	Anthropometric measurement (body weight and height); clinical oral examination and panoramic radiographical imaging	Measuring bite force and masticatory performance evaluation	No difference value of maximum bite force between caries and free-caries group. Caries may reduce masticatory performance in children
4	Gudipaneni et al; The Journal of Clinical Pediatric Dentistry (2020); 44(6): 423–428 16	Children 7–9 years old	60	Assessing the first permanent molars using Caries Assessment Spectrum and Treatment (CAST) index	Measuring maximum occlusal bite-force using movable occlusal force gauze	The maximum occlusal bite force value decrease with the progression of the caries spectrum of first permanent molars
5	Linas et al; Journal of Texture Studies (2020); 51:755–765 11	Children 36–71 months	26	Clinical oral examination using ICDAS, DMFT index, and PUFA index. Occlusion evaluation using IOTN index. Orofacial dysfunction assessment using the NOT-S questionnaire	Mastication evaluation comprised mastication capability and masticatory behavior	The existence of caries could reduce the children's ability to chew, and alter their masticatory behavior
6	Souto-Souza et al; Brazilian Oral Research (2020); 34:e059 13	Children 3–5 years old	384	Clinical oral examination including caries using ICDAS, malocclusion, number of masticatory units. Questionnaire about age, sex, nutritive and nonnutritive sucking habits	Masticatory function evaluation comprised masticatory performance and swallowing threshold	Bottle feeding, posterior malocclusion, and dental caries might impair the masticatory function of preschoolers
7	Barbosa et al; Archives of Oral Biology (2013); 58:1070–1077 7	Children 8–12 years old	150	Dental caries assessment using DMFT index and malocclusion assessment using Dental Aesthetic Index (DAI)	Masticatory performance evaluation and oral health-related quality of life assessment	A higher number of tooth loss correlated with poor MP in older children. Those with more caries rated oral health less favorably. Those who crushed the test material into smaller particles were more likely to report a worse OHRQoL

Abbreviations: BMI, body mass index; DMFT, Decay, Missing, and Filling index; ICDAS, International Caries Detection and Assessment System; IOTN, International Orthodontic Treatment Needs; MP, masticatory performance; NOT-S, Nordic Orofacial Test-Screening; OHRQoL, oral health-related quality of life; WHO, WORLD Health Organization.

To evaluate the masticatory function, the included studies evaluated masticatory performance 12 13 14 and measured the maximum occlusal bite force. 14 15 16 Aside from masticatory performance, Souto-Souza et al also evaluated the children swallowing threshold. 13 A study by Linas et al was the only one that evaluated masticatory function via mastication capability and masticatory behavior. 11 Some studies also evaluated children's food consistency by dietary log that were filled by the parents/caregivers 12 and their OHRQoL. 7

All of these studies found a negative correlation between caries and masticatory function. Although there were no difference value of maximum bite force between caries and caries-free group, caries still might reduce the masticatory performance in children. 14 Meanwhile, other studies found that the maximum occlusal bite force value decreases with the progression of the caries spectrum of first permanent molars. 16 Aside from caries, Consolação Soares et al also stated a negative influence on masticatory performance of preschool children that came from higher frequency of sweet beverage consumption and higher BMI. 12 Another study also stated that a higher number of tooth loss correlated with an inferior masticatory performance in older children. 7 The presence of dental cavity not only interfere with children's chewing ability, but also alter their masticatory behavior, such as food refusal, using tongue to crush food against the palate. 11

2. The cohort studies

Two prospective cohort studies were included in this study with children aged from 2 to 6 years old as the subject. Both studies used children suffering from early childhood caries (ECC) that required treatment under general anesthesia. The study by Collado et al compared any changes with those with no caries, 17 while Khong et al evaluated the dental treatment effect without comparing with a control group. 18

The two studies performed clinical oral examination and caries assessment using the DMFT index. However, Collado et al added several aspect to be explored, such as orofacial dysfunction assessment using NOT-S, orofacial dysmorphology assessment using the IOTN index, and OHRQoL assessment using the Early Childhood Oral Health Impact Scale. 17 The treatment given to children with ECC were multiple teeth extraction, 18 restoration, endodontic treatments, and crown under general anesthesia. 17

Masticatory evaluation was done by assessing interarch functional contact; an elastomer impression was made, and then scanned using image analysis software. A month after treatment, follow-up was performed and found that the deteriorated orofacial function and quality of life caused by ECC were reversible by giving a conservative dental treatment. 17 While the other study that evaluated masticatory function with mixing ability test and assessment of oral processing behavior found the children's mastication function in 3 months after treatment follow-up were unchanged, which was probably due to the loss of masticatory units following the treatment. 18 The summary of the cohort studies is shown in Table 2 .

**Table 2:** The summary of the cohort studies

No.	Authors and journal detail	Subject	Size	Treatment group	Control group	Study exposure	Intervention	Follow-up period	Outcome
1	Collado et al; Med Oral Patol Oral Cir Bucal; (2017); May 1;22 (3):e333-41 17	Children 2–6 years old	41	Children with early childhood caries (ECC)	Free-caries children	- Caries assessment using DMFT index - Orofacial dysfunction assessment using NOT-S - Orofacial dysmorphology assessment using IOTN index, - OHRQoL assessment using ECOHIS - Interarch functional contacts assessment - Number of functional units	Teeth extraction, restoration, endodontic treatments, and crown under general anesthesia for intervention group	1 month after treatment	A conservative treatment was able to restore the altered orofacial functions and quality of life due to ECC
2	Khong et al; International Journal of Paediatric Dentistry;2022;32(3):295–303 18	Children 4–6 years old	25	Children with early childhood caries (ECC)	No control group	- Clinical oral examination - Caries assessment using DMFT index - Masticatory test using mixing ability test and assessment of oral processing behavior - Food preferences evaluated by interview	Multiple teeth extraction under general anesthesia	3 months after treatment	Giving dental treatment did not change the child's mastication function.The increasing number of chews/g following dental treatment was supposed to be associated with the number of posterior extraction

Abbreviations: DMFT, Decay, Missing, and Filling index; ECOHIS, Early Childhood Oral Health Impact Scale; IOTN, International Orthodontic Treatment Needs; NOT-S, Nordic Orofacial Test-Screening; OHRQoL, oral health-related quality of life.

3. The quasi-experimental study

The only quasi-experimental study included in this study observed the differences of occlusal bite force before and 24 hours after treating carious teeth with glass ionomer cement (GIC) restoration ( Table 3 ). The study divided the samples into three groups, caries in all quadrants, caries in upper/lower jaw on the right side, and caries in upper/lower jaw in the left side. Following 24 hours of GIC restoration, they found an increased value of occlusal bite force in all groups. A significant increase of bite force value was also recorded in the free-caries side. 19

**Table 3:** The summary of the quasi-experimental study

No.	Authors and journal detail	Subject	Group division	intervention	outcome measurement	Outcome
1	Subramaniam et al; The Journal of Clinical Pediatric Dentistry 2016;40(4):297–300 19	Children 6–9 years old	Group 1: caries in all quadrants ( *n* 24)	Restoring carious teeth with glass ionomer cement	Recording occlusal bite force before and after treatment	Significant increase of occlusal bite force after restoring primary teeth
			Group 2: caries in upper/lower jaw on the right side ( *n* 8)			
			Group 3: caries in upper/lower jaw on the left side ( *n* 8)			

## Discussion

### Key Findings

Mastication denotes a rhythmical automatic movement, where the food is mixed and crushed with saliva, forming a bolus to be swallowed. It is a complex mechanism involving opening and closing movement of the jaw, saliva secretion, combined with tongue movement to mix the food. 20 The overall finding that can be learned from the included study is caries, either involving anterior or posterior teeth, had influence on the children's ability to chew food. There are various ways available to assess mastication, and the included studies used various objective measures to assess the masticatory function, such as masticatory performance to evaluate the ability to mix test food, occlusal bite force to evaluate muscular activity, interarch functional contacts assessment, mastication ability test, and masticatory behavior assessment to assess the quality of masticatory function. 18 21 22 23

Fortunately, some studies proved that the negative effect is reversible. Applying GIC restoration to the carious teeth were able to increase the value of occlusal bite force. 19 This result is supported by two prospective studies using children suffering from ECC as the subjects, comparing their ability to chew food before and after receiving dental treatment under general anesthesia. 17 18 The studies recorded a successful oral function restoration, including mastication, following dental treatment. 17 While the other study found an increase of chewing difficulty corresponded to the increased total number of extracted teeth. However, the study assumed that the children had ability to adapt to gain the most optimal masticatory function. 18

### Significance of the Findings and Possible Mechanisms

In children, aside from nutritional status, mastication also plays a role in growth process. Studies observing chewing cycle identified well-developed mastication efficiency at the age of 8 months, and for harder solid foods, the earliest maturation identified was at 24 months old. Meanwhile, the studies observing kinematics jaw movement found that by the age of 18 months, children will have well-controlled jaw movement during chewing process. 22 Considering the continuous change of occlusion during primary dentition, in order to illustrate and confirm appropriate growth and development of stomatognathic system, its functional determinants should be established. 19

The oral health state and a particular sensory input from teeth, denotes a decisive key for mastication capability and behavior. Carious lesion progressively affects the tooth structural integrity. Open occlusal or proximal contacts mean losing occlusal force, which also reduce the area to grind the food. Thus, the existence of decayed teeth may alter the children's ability to chew foods with different texture. Besides, children might also modify their masticatory behavior, like extending the chewing time and having chewing-side or certain food preference. Aside from decreasing occlusal force, pain arising from caries also promotes sensitivity during grinding the food. Therefore, whether due to food preference or swallowing bolus in large particles, nutritional consequences should be expected. 11 14 23 Dental caries in children is also at risk of causing preferred chewing side (PCS). 24 If untreated, PCS can develop into an occlusal cant, malocclusion, and cause masticatory disorders, temporomandibular disorder, gingival recession, and alveolar bone resorption. 25 26 27 28

The loss of masticatory units due to the progression of caries can alter the chewing frequency and the bolus granulometry. These factors are responsible for the declining sensory input from the teeth receptors that are required to control muscle and jaw movement during mastication. The adverse effect of caries on maximum bite force associated with input from periodontal tissue mechanoreceptors due to the inadequate occlusal conditions. 14 Although the children had the ability to form mastication behavioral adaptation, the measures were not enough to atone this shortcoming. 11

The reversible effect was proven by two prospective studies and one experimental study. Treating carious lesion means restoring the functional occlusal contact, thus the sensory input from teeth become available again. By restoring the tooth anatomical support, oral function will be recovered, leading to progressing natural occlusal equilibration. 17 19

### Limitations of this Review

The included articles were mostly observational studies, since this study focused on the effect of a disease. This might prevent us to draw the causal inferences between caries and mastication ability. Besides, the included studies use various methods of masticatory function assessment, in various dentition state (primary, mixed, and early permanent dentitions), which makes it quite challenging to conclude.

## Future Research

In order to get the detailed view of the caries effect toward the children's ability to chew, further research can focus on how strong the caries impact on each dentition state. A study that compares the effect of caries on children with primary, mixed, and early permanent dentition group is recommended. Besides, a study that observes a long-term effect of caries during childhood, both treated and untreated, toward mastication ability or behavior in adulthood is also worth considering.

## Conclusion

Finally, it can be concluded that dental caries, specifically the untreated ones, might alter the children's ability to chew. The masticatory function deteriorates as the caries progress. Treating the carious lesion may restore the effect. This finding is expected to be used as reference and principal rationale to give dental treatment as soon as carious lesion is detected.
